# First-year residents’ experiences of uncertainty in rural and urban emergency departments

**DOI:** 10.1186/s12909-026-09021-0

**Published:** 2026-03-20

**Authors:** Sara Gilani, Pål Gulbrandsen, Paul K. J. Han, Marij A. Hillen, Eirik H. Ofstad

**Affiliations:** 1 Health Services Research Unit (HØKH), Department of Research and Innovation, Akershus University Hospital, Lørenskog, Norway; 2 Emergency Department, Nordland Hospital Trust (NLSH), Bodø, Norway; 3 Institute of Clinical Medicine, Faculty of Medicine, University of Oslo, Oslo, Norway; 4 Institute of Clinical Medicine, UiT the Arctic University of Norway, Tromsø, Norway; 5 Division of Cancer Control and Population Sciences at the National Cancer Institute, Maine, USA; 6 Department of Medical Psychology, Amsterdam University Medical Centers (UMC), Amsterdam Public Health, Amsterdam, The Netherlands

**Keywords:** Uncertainty, Resident, Emergency medicine, Young physicians, Doctors, Medical education, Training, Tolerance

## Abstract

**Background:**

Uncertainty is an inherent and challenging aspect of medical practice, particularly for first-year residents making the transition from theoretical education to clinical practice. We aimed to identify factors influencing uncertainty experienced by residents practicing within different organizational frameworks, which could serve as potential targets for supportive interventions.

**Methods:**

A qualitative study was conducted using semi-structured interviews with 20 first-year residents across five organizationally and geographically diverse hospitals in Norway. The reflective interviews were informed by participatory observation during emergency department shifts that provided verbal and behavioral cues signaling uncertainty. Data was transcribed and analyzed using Systematic Text Condensation.

**Results:**

We found four themes with factors influencing residents’ experiences of uncertainty: 1) Residents’ Competence: level of experience and knowledge affected level of uncertainty and confidence, 2) Clinical Complexity: level of patient complexity affected level of uncertainty and the need for consultative support. 3) Consultative Support: availability and predictability of consultative support from senior physician affected level of uncertainty. 4) Level of responsibility: greater responsibility of residents in rural hospitals, due to limited support, fostered faster learning but with the downside of greater initial uncertainty and suboptimal learning environment. We also found that additional factors outside of physicians control such as the number of concurrent patients, the urgency and severity of clinical cases, and related time pressure, further influenced the residents' experience of uncertainty.

**Conclusion:**

First-year residents’ experience of uncertainty is influenced by a combination of personal and organizational factors. Rural hospital settings, which offer limited consultative support and greater responsibility, may foster more uncertainty initially but also promote a quicker transition towards autonomous uncertainty management and development of uncertainty tolerance. Urban settings offer more immediate uncertainty management support, but often under higher patient loads and time constraints, limiting residents’ experience with critically ill patients and responsibility in general. These findings can help hospitals, universities and management modify factors affecting residents’ experiences of uncertainty. This can also help in training medical students and residents so they can better understand, tolerate and manage uncertainty.

**Trial registration:**

This is an observational study which does not include any intervention; therefore, it has not been registered.

**Supplementary Information:**

The online version contains supplementary material available at 10.1186/s12909-026-09021-0.

## Background

Physicians in hospital service make on average more than 13 simple or complex decisions per encounter [1]. Despite substantial advancements in scientific knowledge, uncertainty remains an intrinsic aspect of medical practice [2–5]. As uncertainty borders the edge of knowledge these advancements themselves introduce new forms of complexities. Responding adaptively to the many forms of uncertainty that arise in health care is one of the principal challenges clinicians face [6].

Uncertainty is particularly prevalent among first-year residents [6], who transition from theoretical learning to clinical learning environments in which they have limited experience and ability to make decisions [6–8]. Han et al. define uncertainty as a human epistemic state consisting of the conscious, metacognitive awareness of ignorance [9]. Within the complex system of health care, variability in patient conditions and disease manifestations, along with limitations in clinical evidence, make it difficult to predict patient outcomes [10]. All aspects of clinical work expose residents to uncertainty, from initial information gathering to the formulation of a working diagnosis and the communication of information to patients and colleagues. As residents often work in emergency departments (ED), the uncertainties they encounter take on greater urgency and severity [11, 12], which can further exacerbate the uncertainty they experience. It is well established that uncertainty can have a negative effect on the residents’ psychological wellbeing by increasing stress and burnout [6, 13, 14].

Uncertainty can signal a lack of knowledge, limited clinical experience, limited or conflicting information and challenges in interpersonal communication. Suggesting that reflection and exploration of uncertainty-triggers can help residents learn and grow by understanding their own thoughts and reactions, potentially understanding uncertainty as an important and integral part of medical practice [4, 5].

How individuals manage uncertainty relates to their uncertainty tolerance (UT). Hillen et al. defines UT as “the set of negative and positive psychological responses—cognitive, emotional, and behavioral—provoked by the conscious awareness of ignorance about particular aspects of the world” [15]. UT stems largely from individualistic and cognitive traditions, which may risk underplaying the relational, cultural, and organizational dimensions of uncertainty. First year residents in the ED are often the physicians that see patients first and can experience strong negative emotional responses including insecurity, which may lead to an inability to distinguish clearly between uncertainty as a cognitive state and the feeling of insecurity in response to uncertainty [16, 17]. The latter might lead to overuse of tests or prolonged admissions in the ED and the hospital [18], inability to make decisions, or lack of courage to ask for help.

Professional competence includes the ability to deal with uncertain, challenging situations and make decisions from a limited set of information [19]. In an environment where young physicians are constantly trying to prove their competency, both to themselves and to their supervising physicians, some may choose not to consult a more senior physician when faced with uncertainty as they do not want to be seen as ‘‘weak” or incompetent [10, 19, 20], with possibly negative consequences for the patient [6].

Greater UT and uncertainty management seem to develop over time in practice [8, 9]. Supervisor feedback aimed at stimulating reflection on resident’s practice may promote development of skills for the integration of knowledge and useful management of uncertainties [19, 21] more efficiently. However, residents receive varying degrees of supervision and consultative support depending on local organizational factors [17, 22]. Additionally, individual residents' needs for guidance differ [23–25]. Still, all new residents do need consultative support to manage new medical problems and access to such support varies from one hospital to the next.

There is a lack of studies on uncertainty tolerance and management in clinical settings [12, 26]. EDs are high stake environments with high prevalence of medical uncertainty especially among novice residents [27]. Therefore, we conducted qualitative reflective interviews with each resident following an observed shift in the ED, focusing on their experiences with uncertainty during that shift. We aimed to identify factors influencing uncertainty experienced by residents, which could serve as potential targets for supportive interventions to possibly improve management and tolerance of uncertainty in medical practice. By recruiting and including residents from central and remote hospitals we could take into account potential differences in factors influencing uncertainty at urban vs rural EDs.

## Methods

This paper is part of a larger exploratory qualitative longitudinal study using participatory observation (PO), questionnaires and reflective interviews with 1^st^ year residents in five significantly different emergency departments. In this paper we choose to focus on the rich data from the reflective interviews informed by the PO. The related data from the remaining research methods will be published in following papers.

The COREQ checklist [28] has been consulted to ensure methodological rigor [see Additional file 1].

### Study sites and subjects

During February-April 2022 we recruited 20 residents at commencement of their residency from the five participating organizationally and geographically diverse urban and rural hospitals (Table 1). Two of the hospitals were in southeast Norway, including the biggest and busiest ED in Norway and three were in Northern Norway including EDs on two remote islands. This allowed us to explore potential organizational differences influencing uncertainty experienced by the residents at major urban, medium-sized urban, and smaller rural EDs.

**Table 1:** Approximate numbers from the time of data collection (2022–2023)

	**Hospital 1**	**Hospital 2**	**Hospital 3**	**Hospital 4**	**Hospital 5**
Population the hospital caters to	600,000	60,000	88,000	25,500	22,500
Patient beds in the ED (max)	40	5	13	4	3
Patient contacts in the ED per day (max)	150	40	50	20	20
Physicians present in the ED per shift (max)	12	6	9	4	4
Physicians present in the ED per shift (min)	6	1	5	1	1

The residents’ average work experience prior to residency was 11 months (range 2–45 months, IQR 6–18 months, median 10 months).

### Reflective interviews based on Participatory Observation (PO)

SG, a PhD candidate and medical doctor specialized in psychiatry with clinical work in emergency medicine, shadowed participating residents through PO during two shifts on average 3–6 months apart, each shift followed by a reflective interview. Observational notes were taken during the PO with focus on observed signs of uncertainty. These included verbal and behavioral cues selected by SG, PG and EHO based on relevant uncertainty studies, pilot PO and clinical experience, further described in Additional file 2. The following individual reflective interviews were based on the cues from the notes and a semi structured interview guide [Additional file 3] developed by SG, PG and EHO consisting of standardized questions with added ones based on observations. This method was chosen to allow for open ended questions and reflections throughout the interview enriching the original data without diverting focus from the relevant topics. The interviews were audio recorded, anonymized, and then transcribed verbatim by an external transcriber.

A total of 37 interviews were conducted providing 28 h of relevant data, three of the residents were not able to complete their second interview due to valid reasons for their leave.

### Data analysis

We used Systematic Text Condensation (STC) to analyze the transcriptions. STC is a descriptive and explorative qualitative data analysis method used to identify, code, and categorize themes or patterns in textual data through a structured, step-by-step process [29]. It involves four steps: identifying and coding units of meaning from the data, condensing these units into thematic groups or categories, abstracting the essence of each theme and synthesizing these thematic abstractions into coherent descriptions and concepts. This method was chosen as it allowed intersubjectivity, reflexivity, and feasibility, while maintaining a responsible level of methodological rigor. STC also explicitly prescribes recontextualization as a final step of analysis, where interpretations and findings are validated against the initial complete transcripts [29]. SG identified and coded meaningful units related to uncertainty guided by PG and EHO. Through inductive analysis and with guidance from EHO, the initial condensation process led to ten tentative thematic groups. Data was further condensed by PG, EHO, and SG through an iterative process over multiple meetings resulting in four main themes categorizing modifiable factors influencing the resident’s experienced uncertainty. The relevant citations were translated from Norwegian to English and quality checked by a native English-speaking professional with knowledge of Norwegian. All authors, including PKJH and MH contributed to the discussion of the results.

### Reflexivity

The interpretive nature of our study warranted consideration of the researchers’ positionalities and the ways in which these may have shaped data collection, analysis and the resulting interpretations. Potential subjective influences should be acknowledged and discussed across four domains (personal, interpersonal, contextual, methodological) from planning to manuscript drafting [30]. SG, PG and EHO discussed this in several rounds in the planning phase of the data collection, and SG and EHO spoke regularly throughout data collection period and the data analysis process. SG and EHO’s professional backgrounds and personal experiences, and in particular own relationships to managing and tolerating uncertainty, were identified as important elements to be aware of. SG’s personal, professional and interpersonal subjective influences shaped what was recognized as signs of uncertainty during participatory observation (PO) and interview probing. Aiming to facilitate a neutral ground, the residents had no prior relationship with SG. Addressing the power-dynamic between researcher and subject, the residents were explicitly informed that PO was not an evaluation of their medical practice, and their personal information would be anonymized.

## Results

We identified four themes influencing first year residents’ experiences of uncertainty: 1) Resident’s competence, 2) Clinical complexity, 3) Consultative support and 4) Responsibility.

### Resident’s competence

There are different ways to define and assess competence, in this paper we define individual competence as the sum of medical knowledge and practical skills the resident’s perceived to have relevant to clinical practice in the ED.

Residents' self-perceived levels of competence varied based on where they had attended medical school, the extent and relevance of clinical practice during their education, and the type and amount of clinical work they had engaged in prior to residency.

We identified several sources of inexperience-related uncertainty: unfamiliarity with new medical problems (1a), self-doubt regarding their own competencies (1b-c), hesitancy in seeking adequate help (1a), and fear of making mistakes (1b-c). Moreover, many residents recognized uncertainty as an inherent aspect of medical practice and their training process, with some experiencing an increased tolerance over time (1d) (Table 2).

**Table 2:** Citations related to residents’ competence. Authors have emphasized the text in bold

Finding	Example
1a	*“Yes, I’ve never been here before and have**** little**** orthopedic ****experience…**** And then one ****doesn’t want to bother**** the [consulting physician] too much or ask too many questions. And then one doesn’t get such good… one probably doesn’t get such good training and ****becomes uncertain****.”* -Id3
1b	*“But often one does things that one is ****not necessarily so well trained for**** or like… yes, but one has to, in a sense, do it oneself and try to figure it out a bit along the way in a sense. And it can be a bit ****scary**** sometimes then if one is ****afraid of doing something wrong****”—*Id17
1c	*“I'm a bit ****unsure**** if I've examined well enough, have I pressed hard enough [over the scaphoid bone]? Did I remember to palpate all [necessary structures], and then what was it like?”-*Id12
1d	*"So I think I ****tolerate**** uncertainty better now. But it's also because I feel I have more professional weight. Then there is a big difference between the patients you are ****unsure**** about that you send home than those who must be admitted here. Then you can tolerate that uncertainty to a much greater extent [if the patient is admitted].."—*Id14

### Clinical complexity

As demonstrated in Table 3, the perceived complexity of patients’ clinical problems influenced residents' experience of uncertainty, particularly in severe conditions and cases of rapid deterioration (2a-b). These complex clinical scenarios, coupled with residents' lack of experience, often led to an increased reliance on clinical consultative support, defined as consulting senior physicians (2c). This type of interpersonal support reportedly extended beyond intellectual and cognitive assistance to include emotional and social support, especially when uncertainties were irreducible.

Residents attempted to learn new things despite being uncertain about the complexity of the medical problem and their own inexperience, which could also result in positive feelings when they were successful (2d).

**Table 3:** Citations related to perceived clinical complexity. Authors have emphasized the text in bold

Finding	Example
2a	*“But I think that waiting time is a bit ****unpleasant**** because now the patient is ****not assessed properly****, and I have not managed to find the cause [of his symptoms****]. I don’t understand why**** the patient is so somnolent and lethargic.”…” So this ****wasn’t pleasant.**** “—*Id2
2b	*“But then there is also a ****lot of uncertainty****, you kind of don't know… Like the last patient who asked about that answer to the biopsy and such, and then it's a bit like a non-specific answer… Then you know that the next question will be now what, what do we do next, and then ****I have no idea what we do next****.”–* Id5
2c	*“when we looked at the X-rays, at that thoracentesis [patient], I thought, I'm ****not sure**** of this. Or what is this, I thought. Is it… Could it be pleural fluid? There are so many thoughts. It can be any pathology. But then I knew that I had a senior physician, who can also look at that picture, and then it went really flawlessly, it worked in two seconds; I went there and said can you look at it, and then it was okay. The uncertainty is a bit… It comes every now and then, it does.."–* Id16
2d	*" [first time urinary catheterizing a patient] and then I somehow managed to probe a little and suddenly some large clots came out, and then it was as if it somehow loosened and we [nurse and resident] got it in properly and… So that was a bit of a ****sense of accomplishment**** perhaps, like I thought I wasn't going to get it done at first and then I ****still managed**** it then."—*Id17

### Consultative support

As illustrated in Table 4, our study found that the level of uncertainty experienced by residents in the ED was highly dependent on the perceived availability of consultative support. This access varied widely, ranging from physical presence and active participation in patient examinations to teleconsulting only. Depending on the specific ED and its physician hierarchy, residents could encounter varying levels of availability, ranging from none to multiple senior physicians accessible for consultation. The inability to predict this access exacerbated the uncertainty experienced by many residents.

Across the five hospitals in our study, only the largest ED had a reliable 24/7 physical presence of consulting physicians. However, even in this setting, support could be inconsistent during late evenings and nights due to other duties such as conducting surgeries, participating in trauma teams, or resting/sleeping (3d). Residents reported less access to, and a higher threshold for, seeking consultative support during evening and night shifts (3d). Interactions with senior physicians varied from receiving medical advice and procedural training to engaging in non-conclusive dialogues (3b, d).

Uncertainty was also related to the logistical aspect of the patient’s treatment and transfer to proper departments, which depended on prompt consultations with senior physicians who would make the final decision. Delayed plans for “what to do” and “where to send” the patient often led to the patient staying longer in the ED and subsequently the hospital, affecting the flow of efficiency (3a).

The residents expressed the feeling of inadequacy and shame related to uncertainties that could become a barrier to seeking consultation, even when the support was available (3a, b, d). We also found that residents often had to consult with new and unfamiliar senior physicians during their shifts, which could increase their threshold for when to express their uncertainties and related vulnerability (3c). This was common at the major urban hospital with a bigger team of rotating physicians, but surprisingly also at the smaller rural hospitals due to many substitute physicians.

Our findings demonstrate that residents wanted and needed access to consultative support, either because they did not know how to fully assess and treat the patient (3b), or for the confirmation of their assessment and plan (3a, b, e). Direct access to predictable presence of physical consultative support in the ED was found to be an important factor in uncertainty management (3b, e). Uncertainty would often be alleviated during senior physician consult, which could be pivotal for learning (3e). Conclusively, consultative support influenced both the residents’ perceived uncertainty along with the management of uncertainty.

**Table 4:** Citations related to consultative support. Authors have emphasized the text in bold

Finding	Example
3a	*“It’s a bit because of just the uncertainty, and then it’s a lot because you feel like you’re ****becoming a burden**** on the rest of the emergency team, because it’s so crowded as it is in the ED, and so ****difficult to get patients transferred**** [to suitable wards/treatment]. And then you ****don’t get to fully consult**** about the patient with the senior physician and so you ****don’t get any progress****”—*Id2
3b	*“..[patient] had a humerus fracture, where I ****was only told to put a high cast**** and put it high in the axilla. So, I did it then. But then they were laughing themselves to death, the orthopedic surgeons at the morning meeting, when they saw what I had done. Because it was clearly too far down. So yes, it’s like… it’s the way one feels ****uncomfortable**** when one is freestyling, and then one doesn’t know if what one’s doing is actually good enough, in a way. I think the patients deserve the best. At least when you have the skills in house. When you have orthopedic physicians on the premises, I think that one then should be able to expect that the patients will receive a good cast.”-* Id17
3c	*"Because I feel one doesn't get… it's very rare to get a person [senior physician] one meets several times, especially in the emergency department. And then it becomes a bit ****difficult**** to share uncertainties because yes, because you feel ****vulnerable**** to opening up to strangers." –* Id3
3d	*«To the orthopedists, yes. It is often that… can be evenings where four hours [can pass] without getting in touch with an orthopedist. So it's [difficult]… There is often surgery, surgery, surgery. And then there are times when you just do something you think of. You * ***don't sit with a very good feeling*** *. And then there is… They have a lot to do, but when they operate in addition, it's… they stop by so quickly, and then [they say]… "Do I need to come and look at it * ***or*** *". I know they have a lot to do, but in a way…”.- Id12*
3e	*"And then go and consult a more experienced colleague who may also come in and see the patient ****if I am a little uncertain**** about my findings. So in a way, you get to ****confirm**** what you yourself have found during the examination. It's a bit like that. You are uncertain at first whether you auscultate correctly or palpate correctly. And then that they either agree or slightly disagree with the plan you have thought then. If so, why do they disagree and think that you should rather do it differently…."-*Id9

### Level of responsibility

Finally, residents’ perceived level of responsibility for patient care clearly differed between rural and urban hospitals and was an important determinant of the level of experienced uncertainty. We found that this was organizationally rooted, mainly due to less physical access to consultative support at the rural hospitals (Table 5). Residents in the rural EDs simply did have greater responsibility due to less staff in general combined with expectations from their role and the local culture of care.

During evening, night and weekend shifts the residents at the rural hospitals primarily had access to consultative support by telephone and were alone in the ED. From these hospitals there were large physical distances to more specialized care, resulting in a need for transportation by boat, helicopter, or plane in more urgent cases. Transportation required more time compared to urban hospitals and was dependent on weather, hence patients could also need more urgent care due to transportation delays. This required that residents were able to evaluate an emergency rapidly and do many procedures locally when needed.

We found that residents described the lack of physical consultative support as problematic at times and expressed uncertainty about their own abilities to assess and convey severity of the patient’s emergency (4a-c). They could also experience reluctance from senior physicians when expressing the need for physical consultative support (4b). At the urban hospital, the resident only covered patients admitted to the specialty of their rotation, e.g., surgery. But at the rural hospitals, especially during weekends, the resident received all patients ranging from medical, surgical, orthopedic, psychiatric, pediatric to neurological. They experienced a rapid influx of a wide range of new medical problems and felt greater uncertainty and responsibility for the patient outcome initially but expressed a faster learning curve and more tolerance for uncertainty with time (4c) compared to the urban residents. Access to physical consultative support led to the feeling of safety even during hyperacute and difficult patient trajectories (4d).

In contrast, residents at major urban hospitals had in-house access to physical consultative support for critical patients. This support was often organized within emergency teams, featuring highly trained senior physicians who would promptly respond and assume responsibility.

**Table 5:** Citations related to experience of responsibility. Authors have emphasized the text in bold

Finding	Example
4a	*"…the way I sort it [patient information], the way I present it, has a lot to say for how others [senior physicians] will assess it then. And I think that's a pretty ****big responsibility**** we actually have, when there's no one else who comes and talks to the patient…. And in the beginning, the plan was laid out without anyone [senior physician] else having seen or spoken to the patient. So that then I was ****uncertain**** about whether I had mentioned everything that was relevant, whether I had highlighted the most important things in the medical history/findings" –* Id17
4b	*".. And that perhaps they [senior consultative physicians] have, at least to begin with, believed that we have a higher level of ****competence**** than we do. There have been a lot, especially at the surgical [emergency department] there have been some ****bad experiences**** now since the last time with the new [1st year residents] that started in the fall. Like senior consultants who have not wanted to come [to the hospital] and been like "oh, you can't handle it?" and "you can't do this [yourself]?" and such. And the brand-new residents, straight from the universities who have not been in situations like that before and work alone here at night, have received ****very little support.**** They kind of think that we have to handle everything then, that we must have so much confidence out here? We who are 1 st year residents… So I think that there is not so much double checking and control on everything we do. And it's a bit ****unsafe****. " –* Id13
4c	*" This is a very small hospital. You are very much ****alone**** as a 1 st year resident. You have a ****huge responsibility****, but at the same time… when I talk to other residents at other hospitals, I hear them say that "oh, shit, you are very much alone and with a lot of responsibility and become so ****enormously skilled****." Because it is when you stand alone that you become enormously skilled. You learn so much. But I always think that's a bit difficult, when I haven't done something before, then I get very ****uncertain****… So now when I had a night shift in June, I noticed.. I wasn't so**** scared**** anymore. I was like… Now I know what to ask, and I know how to think. And if I'm uncertain and afraid to take the patient [alone], I can call and ask them [senior consultant] to come. Everyone lives nearby. Should I be very uncertain, they will come. So it has made it easier. And I feel kicked into this, but I've gotten better and learned so much in a short time. From not knowing anything about working in the ED then to, in a way, now when I feel I can in a way have that responsibility. And it's crazy that you can. That it can take only 2 1/2 months before you feel that you are in control. But you are never in ****control,**** but there is enough control that nothing happens, if you know what [I mean]." –* Id16
4d	*"As the procedure is at this hospital, stroke patients are received by 1 st year resident and a senior physician. So that we are a team… one is never alone with a stroke patient. So even though it was ****stressful**** to receive, in this case, two stroke patients that came at roughly the same time, one was never alone. So even though it was a stressful situation, ****I still felt safe****."* [senior physician is called in to the rural hospital by the nurse when a suspected stroke patient is on their way to the hospital]*-* Id15

### Interplay

An overarching question during analysis was how the interplay between these four thematic categories further affected the experience of uncertainty. Both personal and organizational factors played into this. Residents were mostly aware of their own competence levels or limitations, making this a predictable thematic category. In contrast, the complexity of each clinical problem and the availability of consultative support for individual cases were substantially less predictable. However, the level of responsibility residents ended up with was a more predictable element, as they would know if they were working alone at night or with a consultant present 24/7 at the hospital.

**Fig. 1:**
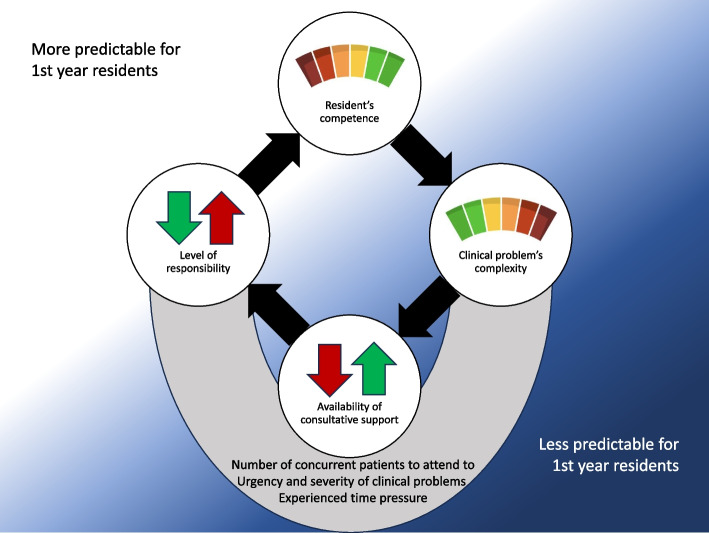
Each theme categorizing factors influencing uncertainty (white circle)—resident’s competence, clinical complexity, consultative support, and level of responsibility—exist at varying levels. We display competence and complexity using gauges, illustrating that these can be high, low or anywhere in between. We display support and responsibility using dichotomous arrows; either it is high, or it is low, and in the case of support, high can increase safety, while in the case of responsibility, high can be frightening. These factors are interrelated, leading to different levels of experienced uncertainty for residents. While competence and responsibility are more predictable (white background), complexity and support are less predictable (blue background). Additional factors further affect this interplay (grey arch), including the number of concurrent patients, urgency and severity of clinical problems and experienced time pressure

As illustrated in Fig. 1, each theme influencing uncertainty—resident’s competence, clinical complexity, consultative support, and level of responsibility—exists at varying levels. Collectively, these influence the degree of uncertainty experienced by residents nationally and internationally and can help develop a framework for managing uncertainty. For instance, residents with low competence may initially benefit from high consultative support and reduced levels of responsibility.

Additionally, factors external to the physician’s and hospital’s control such as the number of concurrent patients, the urgency and severity of clinical cases, and experienced time pressure further influenced the residents' experience of uncertainty (Fig. 1).

## Discussion

Our aim was to identify factors influencing uncertainty, among residents practicing within different organizational frameworks (urban and rural), that could be potential targets for supportive interventions facilitating UT and management. We found four themes categorizing these factors: resident’s personal competence, the perceived clinical complexity, access and quality of consultative support, and level of responsibility. The results show that these categories have an interactive aspect that can further increase or decrease the perceived uncertainty. Figure 1 can help demonstrate this through three examples of combinations: 1. Low Competence, Complex case, High Support, Low Responsibility: Minimizes uncertainty, preferrable for early training stages. 2. Low Competence, Complex case, Lacking Support, High Responsibility: Increases uncertainty, risking high stress and errors, less ideal for training. 3. Medium Competence, Simple case, High Support, Low Responsibility: Low uncertainty but may limit learning experience. By directly addressing uncertainty and level of competence early on with the individual resident, our figure may be pedagogically helpful trying to adapt levels of complexity, consultative support and responsibility to the individual resident. Effectively balancing these elements can be imperative for optimizing the training environment and improving uncertainty management.

In line with other studies [10, 12, 31], our results show that time pressure and concurrent urgent patient load induced anxiety, stress, and a feeling of insecurity. Notably, some residents exhibited comparatively stronger emotional tolerance of uncertainty, also observed in the study by Berenbaum et al. [32]. These residents expressed subsequent feelings of accomplishment and excitement when they managed to learn from the challenges. In rural hospitals, increased responsibility due to limited consultative support heightened uncertainty and led to emotional distress in some residents. Paradoxically, it also led to steeper learning curves and swifter acquisition of practical skills, potentially facilitating faster management of uncertainty and subsequent reduction of anxiety and distress, as supported by other studies [33, 34]. These findings suggest that although reduced support can promote or force faster skill development, it may do so at the cost of increased distress among novice residents in a suboptimal learning arena posing potentially greater risk to patients that are admitted here.

Residents in urban hospitals faced higher patient loads and time constraints, a trend also noted in international studies [35]. Although these residents quickly familiarized themselves with common medical emergencies, they reported less responsibility for decision-making, especially in critical cases. Arguably, lack of responsibility and limited experience from critical emergencies can heighten uncertainty by limiting residents’ experience and learning possibilities.

To pinpoint it, while one could argue that residents in rural settings are left to “sink or swim”, residents at major urban hospitals may end up in “shallow waters with floaties”. Identifying each resident’s “swimming skills” as early as possible is paramount to ensure optimal progression of professional development, while making sure that the residents feel safe during training.

Our impression after these 37 interviews with 20 different residents was that residents with prior experience at their current workplace (through placement during medical studies or clinical work prior to residency) generally reported lower uncertainty regarding practical routines, whereas those without such experience expressed higher uncertainty related to unfamiliar routines further heightening uncertainty experiences, especially in the first months of their residency.

First-year residents’ ability to alleviate and tolerate medical uncertainties appears to be influenced by both personal and systemic factors [17, 26, 32, 36]. These findings suggest several potential ways to improve this ability. For example, hospital management and educators can benefit from introducing new residents to site-specific challenges and assessing their tolerance and management of uncertainty. In a recent study exploring 1 st year residents' responses to five different uncertainty tolerance (UT) measures, Physicians’ Reactions to Uncertainty (PRU) appeared to be a reliable, discriminatory, and feasible measure for identifying junior physicians who are predisposed to stress or other aversive psychological reactions to the uncertainties of clinical care [37]. Simulation and problem-based learning are effective in training for uncertainty tolerance [38] and have been linked to positive cognitive and behavioral responses [39], promoting reflection and constructive feedback [40]. Our results support that organizations, institutions, educators, supervising/consulting physicians should aim to alleviate the extraneous uncertainties through adequate training, senior support and psychologically safe working environments [26]. It is essential to recognize individual strengths and weaknesses as well as needs and preferences, suggesting that personalized training plans could be beneficial [19, 38]. Our findings indicate that ad hoc guidance and consultative support from trained senior physicians are central in understanding and alleviating extraneous uncertainty, facilitating uncertainty management, enhancing learning, and improving wellbeing [6, 19]. The consultative support can range from cognitive/intellectual to emotional/relational and varies throughout. A more methodical approach to investigating uncertainty in health care can help improve clinical communication and management of uncertainty [41]. Finally, uncertainty is and will be an inherent part of the medical profession, facilitating reflection, understanding and innovation. Accepting this can improve tolerance and aid learning [4].

### Strengths & limitations

The strength of this study was that the residents were interviewed by the same researcher who had actively observed and listened to interactions during the shifts, observing for signs of uncertainty and preparing questions that guided the interviews [17, 42]. The researcher’s MD background and experiences increased understanding of what took place in the EDs, but it also introduced the risk of assuming familiarity and overlooking novice perspectives. The chosen methods gave valuable insights connecting the researchers observed cues and the resident’s firsthand reflections as the noted observations could be confirmed, elaborated on or rejected as signs of uncertainty. By recruiting residents from five diverse rural and urban hospitals we were able to discover site-specific factors influencing uncertainty and the longitudinal approach allowed us to identify potential changes in the residents experience of uncertainty and UT with time.

One limitation was that not all interactions could be included due to lack of consent. This happened more often if the patient was critically ill. Even if the patient was able to give consent, when multiple companions/next-of-kin, health personnel, or an emergency team were involved, it was impractical to achieve consent from all. Another limitation was fewer participants from two hospitals compared to the other hospitals, reducing the pool of data for internal comparison and validation. Although the collected data did resemble data from other same-sized hospitals, supporting the overall findings.

## Conclusion

How first year residents experience uncertainty in the emergency department is influenced by various modifiable and interactive person- and system-level factors, particularly resident’s competence, case complexity, consultative support, and assumed level of responsibility. Residents at rural hospitals had less consultative support and felt more responsibility, especially in the evenings and nights, which initially increased uncertainty, but led to steeper reported learning curves and enhanced tolerance for uncertainty with time. Residents in urban hospitals experienced higher patient volumes, got more consultative support and less responsibility for critical cases, which led to less experience with management of critical cases and increased uncertainty. Residents’ experiences with uncertainty may be shaped through gradually adapting levels of complexity, consultative support and responsibility, with the levels of competence. Implementing targeted training programs focused on uncertainty management can mitigate the adverse effects experienced by physicians and in the end, hopefully improve patient care.

## Supplementary Information

Additional file 1. RURRR_A1_Appendix1_COREQ.

Additional file 2. RURRR_A1_Appendix2_Cues.

Additional file 3. RURRR_A1_Appendix3_Iterview_Guide.

Additional file 4. RURRR_A1_Appendix4_STROBE.

## Data Availability

The data that support the findings of this study are not publicly available due to individual privacy and national laws. Further restrictions apply to the availability of the data used under license for the current study. Further details regarding the cues for PO and the semi-structured questions used during interviews can be found in Additional files 2 and 3. Additional file 1 and 4 contain reputed checklists that were consulted while working on the manuscript.

## Abbreviations

ED: Emergency Department
UT: Uncertainty tolerance
COREQ: Consolidated Criteria for Reporting Qualitative Research
PO: Participatory observation
STC: Systematic Text Condensation
IQR: The Interquartile Range
TAMSAD: Tolerance of Ambiguity in Medical Students Scale
PRU: Doctors and Physicians’ Reactions to Uncertainty Scale
