# Supplementary material for: First-year residents’ experiences of uncertainty in rural and urban emergency departments
Source: BMC Med Educ. 2026 Mar 20;26:705. doi: 10.1186/s12909-026-09021-0 (PMC13130759; doi:10.1186/s12909-026-09021-0)
Supplement: Supplementary file 2 — Additional file 2. RURRR_A1_Appendix2_Cues. [file 12909_2026_9021_MOESM2_ESM.pdf]

Observed signs of uncertainty

During PO

Verbal cues

- Verbal pause
- Hesitation, filler words like “um”, “uh”, “like”
- Vagueness, using ambiguous language or generalizations
- Questioning tone, e.g. higher pitch in the end
- Trailing off while talking
- Delayed response
- Slower articulation
- Lack of specific details
- Repeating or paraphrasing
- Elaborate questioning, at times to abstract
- Elaborate explanations
- Asking the patient to repeat information
- Inconclusive answers
- Using medical or non-specific terms that can be difficult for the patient to understand
- Inconsistent statements, contradictory comments and/or changing opinions
- Asking for help/guidance/support
- Using words and phrases like:
    - “maybe”
    - “might be”
    - “sometimes”
    - “I don’t know”
    - “I’m not sure”
    - “I think so”
    - “probably”
    - “who knows”
    - “let me check”
    - “I could be wrong”
    - “Can be this or that”
    - «Can be either/or»

Behavioral cues

- Fidgeting
- Confused look
- Baffled look
- Furrowed brows
- Lack of eye contact
- Shrugging
- Mouth twitch
- Hesitation in movement, hesitant smile
- Pause to note
- Physical pause
- Pause to check literature
- Pause to check for patient history in their journal
- Pause and look away
- Ordering extra tests
- Repetitive examination of a certain area
- Prolonged examination of a certain area
- Longer than average time used on examination
- Overly active use of arms and gesturing
- Protective stance and crossed arms
- Consulting senior physician(s)/others
- Bringing in senior physician(s)
