# Supplementary material for: First-year residents’ experiences of uncertainty in rural and urban emergency departments
Source: BMC Med Educ. 2026 Mar 20;26:705. doi: 10.1186/s12909-026-09021-0 (PMC13130759; doi:10.1186/s12909-026-09021-0)
Supplement: Supplementary file 3 — Additional file 3. RURRR_A1_Appendix3_Iterview_Guide. [file 12909_2026_9021_MOESM3_ESM.pdf]

- Sara Gilani, Pål Gulbrandsen, Paul K. J. Han, Marij A. Hillen, Eirik H. Ofstad

Semi structured interview guide

Reflective interview post-participatory observations (PO)

The interviews were primarily held directly after the PO focusing on observed cues of uncertainty that helped guide relevant questions. The resident’s would return their calling-devices, if possible, to avoid disturbances during the interview, which would mostly last between 45-60 minutes. Following is a list of standardized questions that were used during the reflective interviews with optional questions that could be added. If the resident did not touch upon the topics related to the remaining questions then these or other organic questions could be added during the interview. To get organic data during the interviews the focus was on the residents’ personal reflections on uncertainty and their practice, therefore there was no rigid plan to complete all optional questions or end the interview after 60 minutes, allowing room for flexibility during the interview.

Prioritized questions

1. What are your thoughts on today/tonight’s shift?
2. What are your thoughts on the ED-shifts in general?
3. Do/did you experience any challenges?
4. How do you experience uncertainty?
5. Do you recall any moments during the shift where you experienced uncertainty? If yes, please elaborate.
6. During the consultation with patient xx, I noted xx moment with xx sign of potential uncertainty. If correct; Could you elaborate what you were uncertain about/what you might be thinking during that moment?
7. In xx moment, I saw you did xx, why was that? Could that have been to reduce the uncertainty?
8. Are there certain things/scenarios that help to reduce uncertainty?
9. How do you experience uncertainty now, compared to our first shift together? [Question relevant for the 2nd PO]
10. How do you experience the accessibility of consultative support?

Optional questions based on the individual interview

General

1. What previous medical experience do you have?
2. Is there anything else you'd like to talk to me about concerning the ED? [Relevant towards the end of the interview].

Uncertainty

3. What are your thoughts on uncertainty in clinical practice?
4. During the xx moment of uncertainty, what did you feel?
5. Are there certain things/scenarios that make you more uncertain?
6. How do you handle uncertainty now, compared to our first shift together? [Question relevant for the 2nd PO]

Organizational aspects

1. Did you get sufficient training prior to starting your residency?
2. Do you get sufficient training during your residency?
3. How do you experience the patient load?
4. How do you experience the working environment in the ED?
5. What is your experience of working with the other occupational groups on shift, e.g. nurses, senior physicians?
6. Is there room for organizational improvement? If yes, do you have any (specific) suggestions?
7. Are there any challenges related to consultative support?
8. Do you get adequate support from your senior physicians?
9. Do you get adequate guidance from your senior physicians?
10. Are senior physicians available for consultative support when needed?
11. What is your experience of your responsibilities during the shift(s)?
